# Metabolic and Oxidative Changes in the Fern *Adiantum raddianum* upon Foliar Application of Metals

**DOI:** 10.3390/ijms232314736

**Published:** 2022-11-25

**Authors:** Jozef Kováčik, Lenka Husáková, Petr Babula, Ildikó Matušíková

**Affiliations:** 1 Department of Biology, University of Trnava, Priemyselná 4, 918 43 Trnava, Slovakia; 2 Department of Analytical Chemistry, Faculty of Chemical Technology, University of Pardubice, Studentská 573 HB/D, 532 10 Pardubice, Czech Republic; 3 Department of Physiology, Faculty of Medicine, Masaryk University, Kamenice 753/5, 625 00 Brno, Czech Republic; 4 Department of Chemistry and Environmental Sciences, University of Ss. Cyril and Methodius, J. Herdu 2, 917 01 Trnava, Slovakia

**Keywords:** antioxidant molecules, heavy metals, reactive oxygen species, soil pollution

## Abstract

Cadmium (Cd) or nickel (Ni) were applied as a foliar spray (1 µM solution over one month) to mimic air pollution and to monitor metabolic responses and oxidative stress in the pteridophyte species. Exogenous metals did not affect the metal content of the soil and had relatively little effect on the essential elements in leaves or rhizomes. The amounts of Cd and Ni were similar in treated leaves (7.2 µg Cd or 5.3 µg Ni/g DW in mature leaves compared with 0.4 µg Cd or 1.2 µg Ni/g DW in the respective control leaves), but Ni was more abundant in rhizomes (56.6 µg Ni or 3.4 µg Cd/g DW), resulting in a higher Cd translocation and bioaccumulation factor. The theoretical calculation revealed that ca. 4% of Cd and 5.5% of Ni from the applied solution per plant/pot was absorbed. Excess Cd induced stronger ROS production followed by changes in SOD and CAT activities, whereas nitric oxide (NO) stimulation was less intense, as detected by confocal microscopy. The hadrocentric vascular bundles in the petioles also showed higher ROS and NO signals under metal excess. This may be a sign of increased ROS formation, and high correlations were observed. Proteins and amino acids were stimulated by Cd or Ni application in individual organs, whereas phenols and flavonols were almost unaffected. The data suggest that even low levels of exogenous metals induce an oxidative imbalance, although no visible damage is observed, and that the responses of ferns to metals are similar to those of seed plants or algae.

## 1. Introduction

Increasing industrial and mining activities are unequivocally connected with elevated emissions of various elements. Among them, heavy metals are elements with a density higher than 5 g/cm^3^, which are toxic even at low concentrations and include both essential and non-essential nutrients [1]. Pb, Cd, As or Cr are non-essential whereas nickel is an essential plant “ultramicronutrient” [2]. Heavy metals, including cadmium (Cd) and nickel (Ni), are present in the air [1] and rain [3], so they can easily reach the leaf surface. In some countries, the average Cd content in atmospheric dustfall is increasing [4].

Heavy metals trigger the production of reactive oxygen species (ROS), which is controlled by the synthesis of enzymatic [4,5] and non-enzymatic antioxidants, and nitric oxide (NO) often has a positive effect on the ROS balance [6]. Non-enzymatic antioxidants include mainly general metabolites such as ascorbic acid, glutathione, and phytochelatins, but responses vary depending on the metal/concentration, the duration of exposure, and the species [7,8]. Phenolic metabolites are also abundant plant metabolites with positive health effects that are usually affected by metal excess [9,10]. Metal excess also modulates the accumulation of essential metabolites, such as proteins and amino acids, which are required for the maintenance of enzymatic activities, cellular structures, and the biosynthesis of some antioxidants [8].

Ferns are among the oldest terrestrial plants and therefore their responses to metals may differ from those of seed plants or algae. Although several papers report the accumulation of various metals in ferns growing at different sites [11,12,13], the biochemical responses to metals are less known [14,15]. To our knowledge, antioxidative compounds have rarely been studied in ferns [7,16].

Apart from the relatively limited literature focused on the biochemistry of ferns under metal excess (which we encountered when searching for papers relevant to our discussion), the methods of metal application also vary. The availability of metals in soil is the most common situation but given industrial activities, the presence of metals in the air may represent an additional source of stress. Several studies have reported, for example, the effect of foliar application of metals on epiphytic plants [2], while many other studies have been conducted using terrestrial flowering plants [10,17,18]. For this reason, we selected the common ornamental fern species *Adiantum raddianum* for the present study to investigate the comparative effect of foliar application of Cd or Ni and the use of a dose of 1 µM (equivalent to 11.2 µg of Cd or 5.8 µg of Ni in a single application) over a prolonged period (30 days). Not only metal accumulation but also biochemical changes were monitored to identify specific traits of individual metals. As an original aspect, confocal microscopy and specific staining reagents were used to detect changes in ROS and NO production in the given fern species.

## 2. Results and Discussion

### 2.1. General Parameters

The growth of fern plants and their phenotype were not affected by foliar Cd or Ni application due to the use of bigger plants and a low dose of applied metals (1 µM). Notwithstanding this, tissue water content significantly decreased in both young and mature leaves in response to Cd (from 80 to 76%), supporting previous data that Cd has a more negative impact on water content than Ni [2]. Cd also depletes water content in ferns after longer exposure [7]. No necroses due to metal application were observable.

### 2.2. Accumulation of Ni and Cd Was Comparable in Leaves but Not in Roots

Due to the foliar application of metals, the main part of the solution dripped freely from the leaves (outside the pot) and only a part flowed down the leaf stems into the soil. If the whole applied solution would contaminate soil, it would represent (considering concentration x volume x soil weight per pot) 0.48 mg Cd or 0.25 mg Ni per kg of the soil which would lead to almost doubled soil Cd but only slightly affected soil Ni content. As the solutions mainly dripped freely from the leaves, final contamination of the soil by Cd or Ni did not differ significantly from control soil by the end of the experiment (Supplementary Table S1).

**Figure 1:**
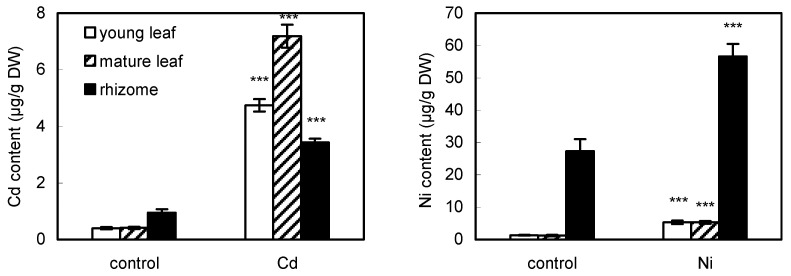
Accumulation of cadmium and nickel in the fern *Adiantum raddianum* after 30 days of foliar application of exogenous Cd or Ni (1 µM solution). Control means plants without exogenous application of metals. Data are means ± SDs shown as bars (*n* = 3) from individual plants/pots. *** indicates a significant difference at 0.001 level of Student’s t-test between control and metallic treatment for the given organ.

The amount of Cd in control plants was ca. 0.4 µg Cd/g DW in leaves and close to 1 µg Cd/g DW in rhizomes (Figure 1). These data are similar to reports from other species such as *Asplenium trichomanes* containing 0.4–0.6 µg Cd/g DW [11], *Pteridium aquilinum* containing ca. 0.3 µg Cd/g DW [12], or *Blechnum orientale* containing ca. 0.2–0.4 µg Cd/g DW [13] in individual organs (fronds or rhizome). In comparison with shoot Cd content in control crops growing in common garden soil (and containing ca. 0.06–0.28 µg Cd/g DW; [9]), it seems that basal Cd content in ferns is not higher than in vascular species. Foliar application of Cd led to ca. 12 or 17-times higher content of Cd in young or mature leaves (in comparison with respective control, Figure 1). The higher amount of Cd in mature leaves may be related to the fully developed blades of individual leaves (leading to the higher surface), whereas the individual blades of young leaves were partially or completely twisted. The absolute amount of Cd reached only 7 µg Cd/g DW in mature leaves though the Cd dose (a total of 1.5 L of 1 µM solution) provided ca. 170 µg Cd per plant/pot: considering ca. 5 g FW of leaves per pot (~1 g DW), it means that ca. 4% from the applied Cd was absorbed. In a similar study where wheat plants were sprayed with a solution containing 10 mg Cd/L, the applied dose (19.8 mL per plant = 198 µg Cd per plant which is similar to our dose) along with Cd amount detected in leaves and stems yielded absorption of ca. 20% from the applied amount (calculable from Li et al. [18]): considering that the difference of Cd amount between control and Cd-treated plants was only up to 3-times in wheat plants (we detected up to 17-fold difference), our data in fern indicate roughly similar uptake from the foliar spray. Accumulation of Cd in rhizomes increased over 3-fold after foliar Cd application (Figure 1), indicating that eventual contamination of soil by Cd was sufficiently absorbed by roots (because soil Cd amount remained unaffected, Supplementary Table S1). Unlike our data where Cd accumulation from foliar spray was negligible, foliar application of Cd (mimicking 1.5–6.5 mg Cd/kg of the dust) led to 22–74 µg Cd/kg DW of rice leaves [19], which is ca. 10-times more than we observed.

The amount of Ni after foliar application increased by ca. 4-times in both young and mature leaves in comparison with the respective control (Figure 1) and the absolute amount of Ni in Ni-treated leaves was similar to that of Cd as mentioned above. In agreement, total leaf Cd and Ni accumulation were similar in an epiphytic vascular plant *Tillandsia albida* after foliar application of 10 µM solutions [2]. Basal leaf Ni content we observed (1.2–1.3 µg/g DW in young/mature leaves) is similar to values reported in *Blechnum orientale* containing ca. 1–2 µg Ni/g DW in fronds [13] but higher values were reported in other ferns such as *Asplenium trichomanes* (21–46 µg Ni/g DW, [11]) or *Pteridium aquilinum* (ca. 4 and 9 µg Ni/g DW in fronds depending on the substrate [12]). For comparison, common crops such as maize or wheat cultured in agricultural soil contained ca. 1.9–4.5 µg Ni/g DW in shoots [9], indicating that the variability of Ni amount in plants is more extensive than that of Cd. The amount of Ni in control rhizomes was ca. 27 µg Ni/g DW and was doubled by foliar Ni application (Figure 1), indicating eventual translocation from leaves to roots: an earlier study reported that Ni was transported mainly to developing organs in three vegetables after leaf contamination while Cd translocation declined strongly with distance from contamination site [17]. The absolute amount of Ni reached ca. 5 µg Ni/g DW in mature leaves though Ni dose (a total of 1.5 L of 1 µM solution) provided ca. 90 µg Ni per plant/pot: considering ca. 5 g FW of leaves per pot (~1 g DW), it means that ca. 5.5% from the applied Ni was absorbed and this value is higher compared to Cd mentioned above.

Although Ni and Cd were applied as a foliar spray, TF (translocation factor = leaf/rhizome ratio of given metal) and BAF values (leaf/soil ratio of given metal) are comparable at least in control treatments with other species and additional elements in all treatments. Basal TF values of Cd and Ni in control plants differ by about 8-fold in favor of Cd (ca. 0.4 vs. 0.05, Supplementary Table S2) due to much higher Ni content in rhizomes (Figure 1). Unlike our data, TF values of Cd and Ni in the fern *Blechnum orientale* were similar (ca. 0.3–1, [13]). Furthermore, *Pteridium aquilinum* revealed similar TF values of Cd and Ni (ca. 0.5–1, [12]), indicating that *Adiantum* fern we analyzed has lower root-to-shoot translocation of these metals. After foliar application of Cd and Ni, respective TF values increased (Supplementary Table S2) but were always higher for Cd owing to lower Cd rhizome content. TF data of other essential macro- and microelements indicated that young or mature leaves accumulated K, Ca, or Cu more than rhizomes (values over 1, Supplementary Table S2) and respective absolute values of individual minerals used for calculation show the same (Supplementary Table S3). The impact of Cd or Ni on TF values of essential nutrients revealed a non-significant impact in young leaves and only Mg or Cu were slightly affected in mature leaves (Supplementary Table S2). Among macronutrients, potassium was the most abundant, followed by magnesium and calcium in all organs (Supplementary Table S3) which is similar to other plants [9]. Foliar Cd or Ni application had no effect and only Mg decreased slightly in rhizomes (Supplementary Table S3). The order of essential micronutrients was Fe > Mn > Cu, which is consistent with other fern species such as *Asplenium trichomanes* [11] or *Pteridium aquilinum* [12]. Mature leaves were more sensitive to foliar application of Cd or Ni where Cu and/or Mn decreased (Supplementary Table S3). In agreement, foliar application of 10 µM Cd or Ni on the leaves of the epiphytic vascular plant *Tillandsia albida* had negligible impact on mineral elements and only Cu decreased [2].

The amount of Cd in the garden substrate we used for cultivation was similar to common garden soil analyzed previously (0.34 µg/g soil) but Ni content was much lower compared to the given soil (42 µg/g, [9]). At the same time, the amounts of both Cd and Ni in our substrate were similar to those found at different sites in China where the fern *Blechnum orientale* was growing (up to 0.49 µg Cd and 12.1 µg Ni/g soil; [13]). For this reason, BAF values (bioaccumulation factor, ratio of leaf/soil metal content) were higher for Cd (ca. 0.8 in controls) than for Ni (ca. 0.1 in controls, Supplementary Table S2) and similar values are calculable in the mentioned *Blechnum* (ca. 0.13–0.27 for Ni and 0.75–1 for Cd, [13]). Furthermore, *Asplenium trichomanes* shows higher BAF values for Cd than for Ni (calculable from Pavlova and Karadjova [11]) so it seems to be a general phenomenon that ferns take up more Cd than Ni with respect to total soil content. Foliar application, therefore, elevated more BAF of Cd (ca. 10-times) than of Ni (ca. 4-times). BAF values of K, Ca, and Mg in young and/or mature leaves were over 1, indicating that all these elements are actively accumulated in the given fern species (Supplementary Table S2). Similar behavior of K and Ca BAF values were also detected in crop species [9] so it is a more general trend in vascular species. The subsequent impact of foliar Cd or Ni application on the BAF of individual elements revealed a negative impact on Cu and Mn, which is in line with the depletion of their absolute values (cf. Supplementary Tables S2 and S3). However, the overall quantitative changes of mineral nutrients do not show an extensive negative impact of foliar metals on the balance of minerals.

### 2.3. Oxidative Stress Evoked by Cd and Ni Differed

**Figure 2:**
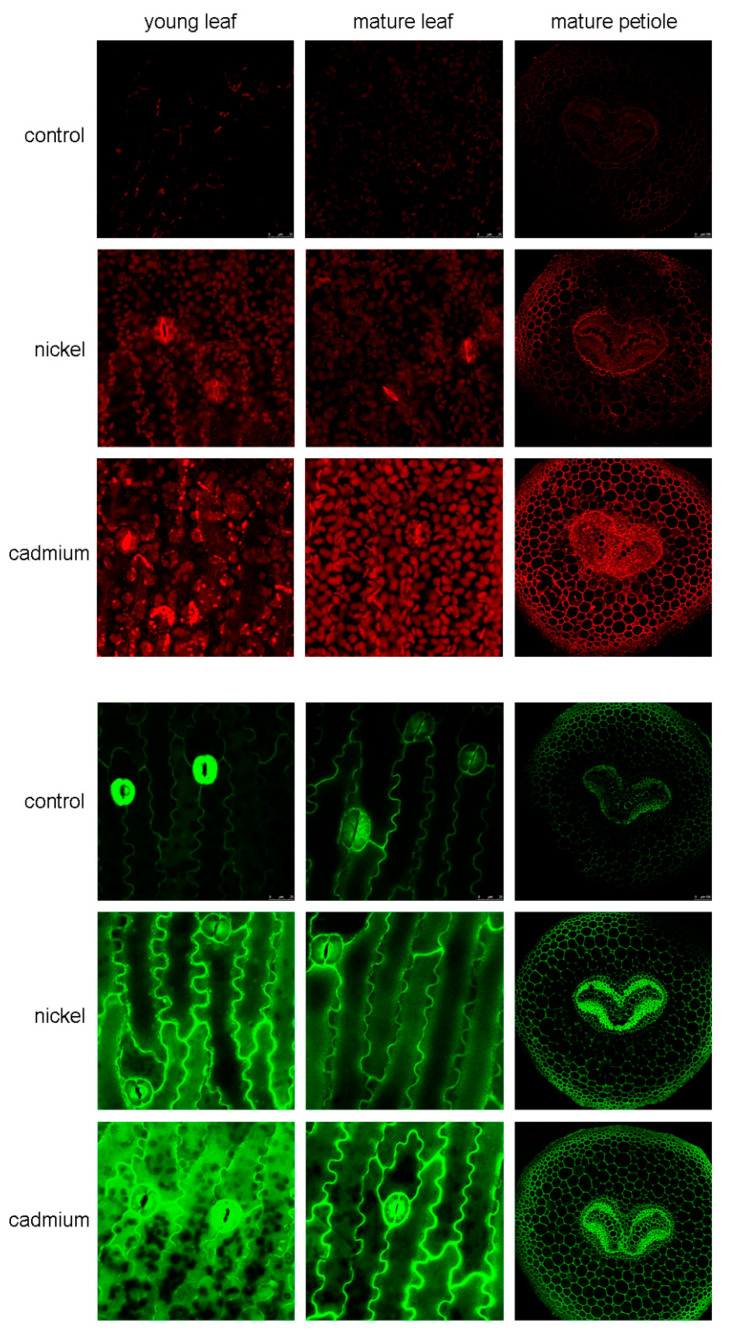
Formation of the reactive oxygen species/ROS (red signal, visualized using CellROX Deep Red Reagent) and nitric oxide/NO (green signal, visualized using 4-amino-5-methylamino-2′,7′-difluorofluorescein diacetate) on the abaxial side of young or mature leaf blades or on the cross-section of mature petiole in the fern *Adiantum raddianum* after 30 days of foliar application of exogenous Cd or Ni (1 µM solution). Bar indicates 20 µm. Note the presence of a hadrocentric vascular bundle in the petiole that resembles the “butterfly wings” typical of ferns. See Supplementary Figure S1 for relative ROS/NO fluorescence.

Metals typically stimulate the formation of reactive oxygen species (ROS) which may be harmful if generated in excess. The formation of metal-induced ROS is often concentration-dependent but the methods (qualitative vs. quantitative) reveal various sensitivities [20]. Higher ROS formation in response to Cd than to Ni was detected both quantitatively (spectrophotometry) and qualitatively (fluorescence microscopy) in vascular plants or in algae [2,8]. In line with these data, mainly mature leaves gave higher ROS signals under Cd treatment in comparison with respective Ni treatment or young Cd-exposed leaves (Figure 2 and see Supplementary Figure S1 for relative ROS fluorescence). It is also interesting that ROS signal under Ni excess did not differ considerably between young and mature leaves: all these observations fit well with the quantification of metals (cf. with Figure 1) and confirm that even subtle changes of metal content are reflected in the altered ROS formation. Cross-section of mature petiole also confirmed a higher stimulatory impact of Cd than of Ni on the ROS signal (Figure 2) and may also indirectly confirm that metals were transported, at least partially, from leaves to rhizomes because the ROS signal increased in petioles and accumulation of metals increased in rhizomes (cf. Figure 1 and Figure 2). Confocal microscopy is one of the most accurate devices for both horizontal and vertical resolution, and the images clearly show that the source of the ROS signal was mainly chloroplasts (see the surface of individual blades of young and mature leaves in Figure 2). On a cross-section from the petiole, an increase in ROS signal was visible in the basal parenchyma and especially in the vascular bundle, which in ferns is referred to as hadrocentric and has the appearance of “butterfly wings”.

Nitric oxide is a small gaseous molecule having numerous physiological functions in plants. To our knowledge and based on the search through the Scopus database (keywords fern and nitric oxide), we found no direct study reporting the impact of abiotic stress on the NO formation in ferns. The only recent study detected NO signal (with confocal microscopy and a similar staining reagent as we used) in the guard cells of the fern *Matteuccia struthiopteris* [21] and, in line with these data, our photos revealed the presence of NO in the guard cells and adjacent cuticle (see “wavy lines”) in the control treatment (Figure 2, green signal). Metallic treatments (Cd and Ni) increased NO signal both in young and mature leaves if compared with control while differences between metals were rather low mainly in mature leaves: Cd in young leaf induced the most pronounced impact and chloroplasts as dark spots were also visible (Figure 2 and see Supplementary Figure S1 for relative NO fluorescence). In this case, the most pronounced NO signal was observed in the vacuoles of unspecialized cells. In the case of stomata, a relatively weak NO signal was observed in the vacuoles of guard cells (compared to the control), but on the contrary, a very strong one was observed in the cell wall. Data clearly suggest that (unlike the ROS detection mentioned above) the NO staining reagent we used visualize also the cuticle so either the limited penetration of the staining reagent or the specific source of NO in the cuticle is plausible. Notwithstanding this uncertainty, the cross-section of petiole confirmed observations from leaf blades, i.e., enhanced NO formation in response to both metals, mainly in ground tissue and primary phloem (Figure 2, right green column) although the differences from the control were less intense compared with ROS (as also confirmed by relative fluorescence quantification, see Supplementary Figure S1). Owing to the overall positive impact of NO on the metabolic changes upon exposure to metals including Cd [6], higher NO formation observed here may be a sign of stimulation of protective mechanisms in response to low exogenous stress. In line with the present data, algae exposed to the same 1 µM Cd dose over 30 days revealed an unaltered NO signal and maize exposed to 1 µM Cd over 10 days showed higher ROS and NO formation [5,20]. It, therefore, seems that NO metabolism in fern is similar to non-vascular and vascular species exposed to Cd excess (low, i.e., environmentally relevant doses).

Changes in ROS formation may naturally originate from the changes in the activities of antioxidative enzymes. Depletion of SOD was observed in both mature and young leaves in response to Ni and more to Cd, which may result in excessive ROS formation, while CAT activity differed (Supplementary Figure S2). Just elevated CAT activity in the young leaves may be, at least partially, the reason for less extensive ROS formation in the young leaves (cf. Figure 2 and Supplementary Figure S2). Additionally, fern *Dryopteris filix-mas* showed elevated CAT activity when cultured with Ni doses up to 1500 mg Ni/kg soil [22]. Furthermore, *Brassica campestris* responded to increasing foliar application of Cd (150–500 µg/m^3^) by a mild but significant increase in the CAT activity and MDA accumulation, and simulated acid rain further enhanced these responses [4]. Relatively high basal SOD values were observed in the species of the genus *Pteris* but arsenic had a rather negligible impact on its activity [23].

### 2.4. Antioxidative Metabolites Are Variously Affected by Cd and Ni

**Figure 3:**
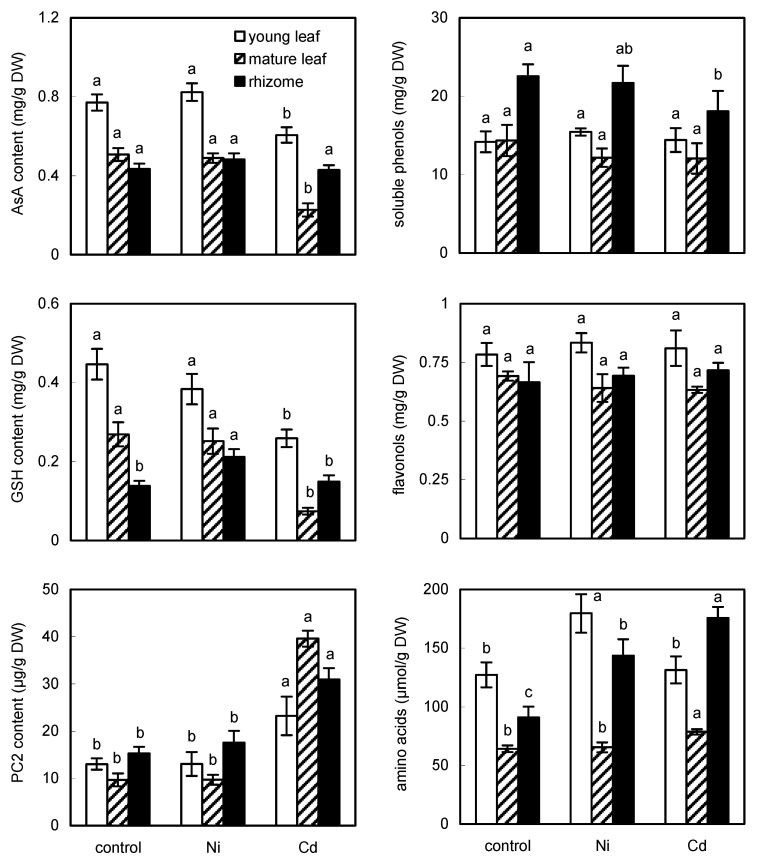
Accumulation of ascorbic acid (AsA), reduced glutathione (GSH), phytochelatin 2 (PC2), phenolic metabolites, and free amino acids in the fern *Adiantum raddianum* after 30 days of foliar application of exogenous Cd or Ni (1 µM solution). Control means plants without exogenous application of metals. Data are means ± SDs shown as bars (*n* = 3). Columns for individual organs, followed by the same letter(s), are not significantly different according to Tukey’s test (*p* < 0.05).

The amount of ascorbic acid (AsA) differed between organs (Figure 3) and the absolute value was higher than that in the moss species but lower than in vascular species assayed by the same method [24], confirming evolutionary differences. The highest reported value here (control young leaves) corresponds to ca. 150 µg if expressed per g FW: reports on the AsA quantity in ferns are rather rare but the published values differ strongly, e.g., control treatment of water fern *Salvinia natans* contained only ca. 1 µg AsA/g FW (or ~5 µg of total AsA/g FW, [16]) while common species of the genus *Polypodium* or *Dryopteris* contained ca. 300–1300 µg AsA/g FW [25], indicating that the AsA quantification in ferns requires further analytical investigation. In line with the more negative impact of Cd on the ROS balance (i.e., enhanced ROS formation), the amount of AsA decreased in the leaves (Figure 3), although no visible signs of damage were observed. In contrast to our results, short-term Cd treatment increased the amount of AsA in aquatic plants [24] or in water ferns [16], suggesting that the long-term impact we tested had a more negative effect.

The same was observed at the level of GSH accumulation, i.e., depletion in response to Cd excess in the leaves while responses of AsA and GSH to metals differed from those in the rhizome (Figure 3). In line with our results, Cd excess depleted GSH quantity in the water fern *S. natans* [16] whereas GSH amount in the shoots of fern *Athyrium wardii* cultured in the soil with Pb excess over 40 days increased [14]. In terms of the experimental setup, foliar application (as we did here) also depleted the GSH amount in the epiphytic vascular plant *Tillandsia* [2]. Phytochelatins (PCs) are metal-chelating compounds with various degrees of polymerization [16] synthesized from GSH so the depletion of GSH in response to metals is often accompanied by an increase in PCs as previously observed in Cd-exposed algae whereas the effect of Ni was not the same [8]. In line with this general trend, Cd stimulated an increase in PC2 in all organs (Figure 3) and the correlation between GSH and PC2 was negative in leaves but not in the rhizome (Supplementary Tables S4–S6). Unlike our data, water fern *S. natans* did not produce a higher amount of PCs after exposure to Cd or multimetallic treatment [16] while responses to soil Pb excess in the fronds of *Athyrium wardii* varied in relation to ecotype [14]. However, correlation analyses also revealed that (using all organs) the relation between Cd and PC2 is positive (r = 0.5809) but Cd and GSH had a negative correlation (r = −0.5335) and this is a typical response of plants (including algae) to Cd excess. We note, however, that the applied dose we used (1 µM) is low compared to many other studies so it seems that quantitative changes of thiols in ferns are sensitive as in the case of algae [8].

### 2.5. Cd Had More Pronounced Impact on Primary and Secondary Metabolites

Soluble proteins are often negatively affected by the excess of metals but the present data revealed their stimulation in response to Cd excess (Supplementary Figure S2). In line with this observation, Cd also evoked an increase in free amino acids in mature leaves while Ni in the young leaves and both metals stimulated the accumulation of amino acids in rhizomes (Figure 3). In line with the present findings, even 2 months of foliar application of 10 µM Cd or Ni on the epiphytic plant had no negative impact on proteins and Ni even stimulated the accumulation of amino acids [2]. In the water fern *S. natans*, Cd excess ca. doubled proteins [16] while arsenic depleted free amino acids by ca. 50% in the fern *Pteris cretica* [15]. In terms of quantity, a lower amount of proteins has been reported in ferns (in comparison with seed species), e.g., ca. 0.2 mg/g FW in *S. natans* [16] and our value would be ~0.36 mg/g FW in control leaves. Similar content of amino acids as we found (ca. 25 µmol/g FW = 127 µmol/g DW in control young leaves, Figure 3) has been observed in the fern *Pteris cretica*, ca. 12 µmol/g FW [15]. These data suggest that a low dose of stress may stimulate the metabolism of nitrogenous metabolites, which contribute to the stabilization of cellular structures, enzymatic activities, GSH/PC biosynthesis, and others. Assay of inorganic N showed that Cd doses of 5–40 µM depleted the amount of N in leaves but not in roots of aquatic fern so the sensitivity may also vary with respect to terrestrial/aquatic environments [26].

Phenolic metabolites are essential plant antioxidants and the same valuable effect is important as a part of the human diet. The basal amount of soluble phenols in leaves (ca. 14 mg/g DW, Figure 3) is rather similar to vascular species than to moss assayed by the same method [24]. Furthermore, four fern species including common *Pteridium aquilinum* contained ca. 9–18 mg of soluble phenols/g DW [27]. It was therefore surprising to find that *Pteridium aquilinum* and many other species contained 82—over 300 mg of phenols/g DW [25] and we have doubts about this quantity simply because even green tea does not contain tens of phenolic%. The amount of flavonols was much lower compared to soluble phenols, as previously reported in vascular species [2] or in other ferns [27]. Following foliar metal application, flavonols in all organs and phenols in the leaves remained unaffected (Figure 3): foliar application of Cd on epiphytic plant slightly stimulated flavonols [2] while foliar application of Ni (0.312 mg/L = ~5 µM) on the medicinal plant *Calendula officinalis* stimulated phenols but depleted flavonoids [10]. Various species, therefore, respond variously to exogenous metals but the absolute change has never been extensive. Data also indicate that phenolic metabolites were almost unaffected in our experiment so the relatively low exogenous stress we applied does not stimulate their accumulation and no signs of visible damage were observable.

### 2.6. Correlation and PCA Analyses

Correlation analyses in young leaves, mature leaves, and rhizomes (Supplementary Tables S4–S6, respectively) revealed differences between parameters in individual organs. Positive correlation between AsA and GSH and negative correlations between PC2 (phytochelatin 2) versus AsA or GSH have been observed in both young and mature leaves (Supplementary Tables S4 and S5) and confirm a balanced biosynthesis of individual antioxidants in response to metals. Significant and both positive or negative correlations have also been observed between antioxidants (AsA, GSH, or PC2) and antioxidative enzymes (CAT and SOD) or metabolites (free amino acids and proteins) in young or mature leaves (Supplementary Tables S4 and S5). In the rhizomes, the PC2 was positively correlated with free amino acids (Supplementary Table S6), indicating that some amino acids are a source for GSH and the subsequent biosynthesis of PCs. It was interesting to find that PC2 was negatively correlated with some elements such as Fe, Cu, or Mg in individual organs: negative correlations have also been observed between mineral nutrients and some metabolites or enzymes but only some of them were significant (Supplementary Tables S4–S6).

**Figure 4:**
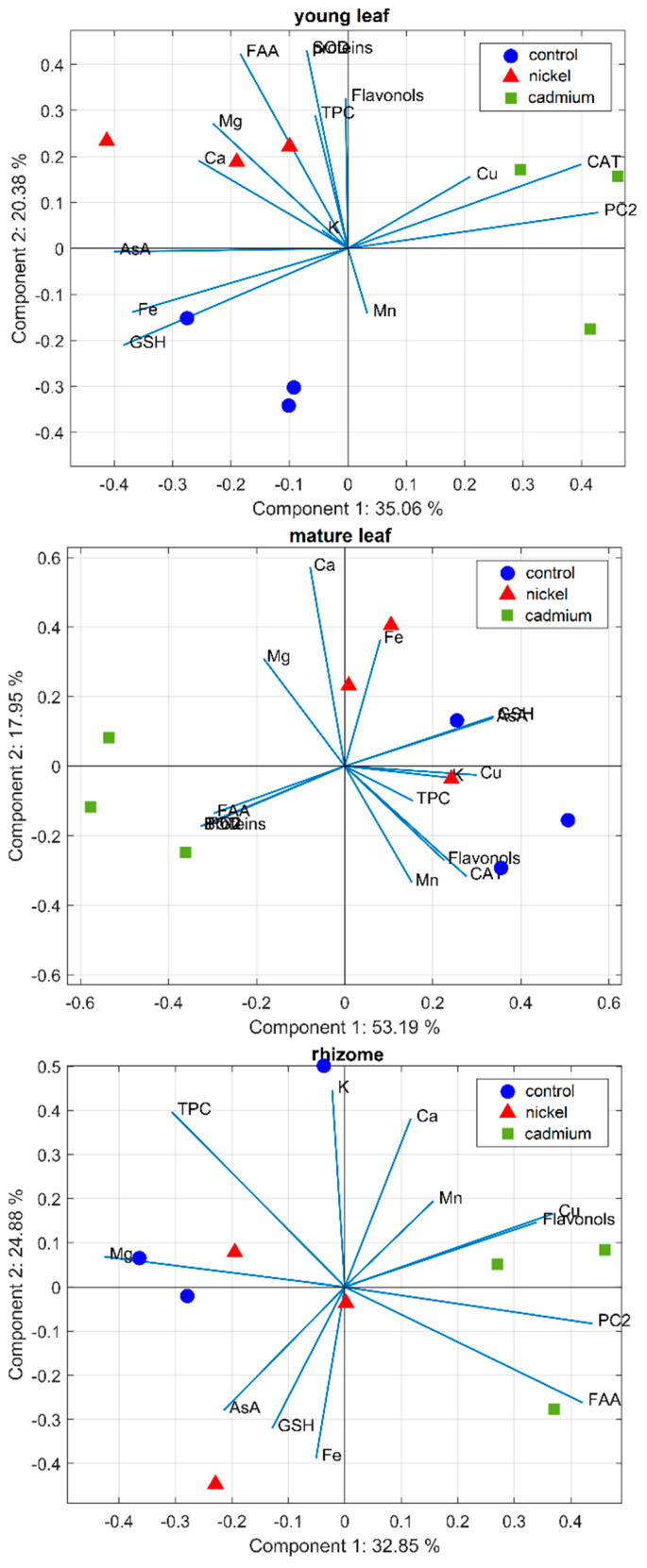
Biplot illustrating PCA analyses of selected parameters in the individual organs of the fern *Adiantum raddianum* after 30 days of foliar application of exogenous Cd or Ni (1 µM solution). TPC—total phenolic content (meaning soluble phenols), FAA—free amino acids, AsA—ascorbic acid, GSH—reduced glutathione, PC2—phytochelatin 2, CAT—catalase, SOD—superoxide dismutase.

The PCA biplots were prepared for the data of young and mature leaves and rhizomes to gain an in-depth understanding of the role of the individual analytes (Figure 4). A biplot overlays a score and loadings plot in a single graph, where the points and vectors are the projected observations and variables, respectively. The samples close to the variables (analytes) represent the correlation between the samples with respect to their level of elements. All of the samples lying on the left side of the axes indicate a minimum concentration of analytes compared to those on the right side of the axes. The initial investigation revealed that, in all cases, three main groups have been identified in PCA biplots, represented by samples of leaves treated with Cd and Ni and control ones. In the model, 55, 71, and 58% of the total variance in young and mature leaves and rhizomes could be explained by PC-1 and PC-2, respectively.

For young leaves, Figure 4 revealed that the predictive variables phytochelatins 2 (PC2), CAT, AsA, GSH, or Fe contribute heavily to the first principal component separating samples of leaves treated with Cd from controls or samples exposed to Ni, while SOD, proteins, FAA, flavonols, TPC or Mg contributes the most toward PC-2 and to the separation of by Ni-treated samples. More detailed information can be found in Supplementary Figure S3 visualizing the contributions of each variable to the principal components as a heat map. Associated with mature leaves, the characteristics that contributed the most for PC-1 were proteins, SOD, GSH, AsA, PC2, or FAA whereas, for the second component, the largest contributors were variables Ca, Mg, Fe, Mn, or CAT (see Figure 4 and Supplementary Figure S3). The PCA biplot (Figure 4) and the parallel coordinate plot (Supplementary Figure S4) revealed that mature leaves of *Adiantum raddianum* under Cd treatment had the lowest values of AsA, GSH, CAT, or Cu, higher levels of proteins, FAA, SOD, and PC2, while mature leaves treated with Ni had elevated levels of K, Fe and Ca. What can be further seen from Figure 4 and Supplementary Figure S4 is that the highest scores for rhizome samples treated with Cd show a higher concentration of Cu, flavonols, FAA, or PC2, while elevated levels of GSH and AsA slightly differentiate a group of samples treated with Ni.

## 3. Materials and Methods

### 3.1. Plant Material, Cultivation, and Experimental Design

Plants of the fern *Adiantum raddianum* were purchased from a garden shop and about 3 cm large fragments of rhizomes with a few mature leaves were planted in individual pots containing common gardening substrate (AGRO CS, Lučenec, Slovakia) with (in dry mass) 1.9% N, 0.5% P and 0.9% K, organic mass 70%, pH 6.0 and humidity of 65%. One 9 cm diameter pot contained 350 g of soil (dry weight) and the whole cultivation was carried out under artificial light with photosynthetically active radiation (PAR) ~200 µmol.m^2^.s^−1^ (cool white light, Osram Lumilux 36 W tubes, Osram, Munich, Germany) in a cultivation room with a day length of 12 h and a temperature of about 25/20 °C day/night. The substrate contained (as pseudo-total content) 0.55 µg Cd and 12.1 µg Ni/g DW.

After 4 months of adaptation, when plants had approximately 15 mature leaves (dark green with fully developed leaf blades) and approximately 20–25 young leaves (light green without fully developed blades), foliar spraying was initiated by applying 1 µM Ni or Cd (in the form of chloride dissolved in deionized water) using a commercial hand sprayer and a volume of 100 mL per plant/pot. Plants were sprayed each 48 h over 1 month, leading to 15 applications and 1.5 L of solution per plant/pot. Control plants were sprayed with an identical volume of deionized water only. The soil was not protected against droplets to mimic realistic natural conditions and volume was applied in two doses within several hours (notwithstanding this, the main part of the solution dripped freely from the leaves out of the pot). One pot was considered as one biological replicate. After the exposure period, rhizomes were carefully removed from the soil and washed with deionized water, and leaves were also washed and dried with filter paper prior to extraction for parameters requiring fresh material. Parallel dry samples (dried at 75 °C to constant weight) were used for the quantification of minerals, phenols, and free amino acids, and water content was calculated [% = 100 − (dry mass × 100/fresh mass)]. Processing of dry samples for the assay of metabolites involved manual extraction with mortar and pestle and with the addition of so-called inert “sea sand” to obtain complete tissue disruption [28].

### 3.2. Quantification of Metals and Minerals

Dry plant samples were mineralized in the mixture of 16% HNO_3_ and 30% H_2_O_2_ (5:2) and soil samples in *aqua regia* containing 37% HCl and 65% HNO_3_ (7:2.5) using a microwave oven, speedwave XPERT (Berghof, Eningen, Germany). All measurements were performed using an Agilent 7900 ICP-MS equipped with an octopole-based collision/reaction cell to remove polyatomic interferences. Non-spectral and matrix interferences were minimized using an internal standard solution containing 200 µg L^−1^ Rh, introduced in parallel with the analyzed solutions. The plasma and instrument operating conditions, together with the validation parameters of the employed analytical method were the same as reported previously [28].

### 3.3. Assay of Metabolites and Enzymes

Reduced ascorbic acid was extracted from fresh material in 0.1 M HCl and quantified by the bathophenanthroline method [5]. Thiols (reduced glutathione and phytochelatin 2) were extracted and quantified by derivatization with monobromobimane by HLPC as reported previously [29].

To detect enzymatic activities of catalase and superoxide dismutase, fresh tissue was extracted in 50 mM K-phosphate buffer containing 1% insoluble PVPP (pH 7.0) and monitored by spectrophotometer, including the quantification of soluble proteins by Bradford’s reagent [5]. An assay of free amino acids was performed with the ninhydrin method following extraction in 60% aqueous ethanol [28].

Total soluble phenols and flavonols were quantified in 80% aqueous methanol extracts with Folin–Ciocalteu phenol reagent or AlCl_3_ reaction as described in detail previously [28].

### 3.4. Microscopic Analyses

For the visualization of general ROS, CellROX Deep Red Reagent (Life Technologies, Carlsbad, CA, USA) was used as a 5 µM working solution prepared in 50 mM phosphate-buffered saline (PBS, pH 6.8) with 60 min staining period at 37 °C. Nitric oxide was visualized by 4-amino-5-methylamino-2′,7′-difluorofluorescein diacetate (DAF-FM DA) by using 50 µM working solution in PBS (50 mM, pH 7.2) and 60 min staining period at room temperature as reported previously [8,20]. Samples were washed with respective buffers prior to observation by confocal microscope Leica TCS SP8 X (Leica, Wetzlar, Germany). Images were processed using the NIS Elements software (Nikon, Tokyo, Japan), including the relative quantification of ROS and NO as an average intensity.

### 3.5. Statistics

Statistical analysis was performed using MINITAB Release 11 (Minitab Inc., State College, PA, USA) and MATLAB^®^ R2022a (The MathWorks, Inc., Natick, MA, USA) software. Data were evaluated for normality and homogeneity using the Shapiro-Wilk and Levene tests, respectively. If the normal distribution and/or homogeneity of variance assumptions were violated, the Box-Cox normalizing and variance stabilizing transformation was employed. Although a simple *t*-test was used to compare the mean of one group with another, analysis of variance (ANOVA) compared the means of several groups to test the hypothesis that they are all equal, against the general alternative that they are not all equal. In this case, a Tukey test performed multiple pairwise comparisons for all distinct pairs of groups. Pearson’s correlation analysis was used to calculate the pairwise correlation coefficient matrix with the corresponding significance test *p* values, between all pairs of variables. In all cases, a *p*-value lower than 0.05 was selected as the statistical significance threshold. Finally, a PCA analysis was used to find the directions of maximum variance in high-dimensional data and project it onto a smaller-dimensional subspace while retaining most of the information to identify patterns in the data.

## 4. Conclusions

The present study confirmed that a relatively low exogenous metal dose (1 µM) with repeated application affects the oxidative balance as detected by ROS and nitric oxide formation using confocal microscopy. Even such a gentle metal dose modulated the antioxidants (ascorbic acid, glutathione, phytochelatin 2) and antioxidant enzymes aimed at providing protection against oxidative imbalance, and no visible signs of damage were observed. Cadmium evoked more extensive changes than Ni although theoretical calculation revealed that ca. 4% of Cd and 5.5% of Ni from the applied solution per plant/pot was absorbed. The data also suggest that the responses of ferns to metals are similar to those of seed plants or algae, and PCA analyses clearly separated the effect of Cd from that of Ni.

## Data Availability

The data presented in this study are available on request from the corresponding author.

## Supplementary Materials

The following supporting information can be downloaded at: https://www.mdpi.com/article/10.3390/ijms232314736/s1.
