# Supplementary material for: Metabolic and Oxidative Changes in the Fern Adiantum raddianum upon Foliar Application of Metals
Source: Int J Mol Sci. 2022 Nov 25;23(23):14736. doi: 10.3390/ijms232314736 (PMC9740585; doi:10.3390/ijms232314736)
Supplement: Supplementary file 1 [file ijms-23-14736-s001.zip › ijms-1943656-supplementary.pdf]

Supplementary materials for the work:

Metabolic and oxidative changes in the fern *Adiantum raddianum* upon foliar application of metals

Jozef Kováčik [1,*], Lenka Husáková [2], Petr Babula [3], Ildikó Matušíková [4]

[1] Department of Biology, University of Trnava, Priemyselná 4, 918 43 Trnava, Slovak Republic
[2] Department of Analytical Chemistry, Faculty of Chemical Technology, University of Pardubice, Studentská 573 HB/D, 532 10 Pardubice, Czech Republic
[3] Department of Physiology, Faculty of Medicine, Masaryk University, Kamenice 753/5, 625 00 Brno, Czech Republic
[4] Department of Chemistry and Environmental Sciences, University of Ss. Cyril and Methodius, J. Herdu 2, 917 01 Trnava, Slovak Republic
* Correspondence: jozkovacik@yahoo.com (J.K.); Tel.: (+421 33 5939 519), https://orcid.org/0000-0002-5701-0598

Supplementary Table S1. Amount of selected macronutrients and micronutrients **in the soil** at the end of the experiment with foliar application of Ni or Cd on the leaves of *Adiantum raddianum* during 30 days (1 μM solution). nd – not determined. Data are means ± SDs shown as bars (*n* = 3) from three individual pots. Values within rows, followed by the same letter, are not significantly different according to Tukey's test (at *P*<0.05 level).

	control	Ni	Cd
Ni (μg/g DW)	11.8 ± 2.90 a	12.4 ± 3.56 a	nd
Cd (μg/g DW)	0.47 ± 0.06 a	nd	0.51 ± 0.09 a
K (mg/g DW)	3.52 ± 0.38 a	3.61 ± 0.27 a	3.75 ± 0.29 a
Ca (mg/g DW)	2.61 ± 0.27 a	2.79 ± 0.34 a	2.87 ± 0.24 a
Mg (mg/g DW)	4.57 ± 0.18 a	4.33 ± 0.15 a	4.26 ± 0.20 a
Fe (μg/g DW)	10549 ± 767 a	11301 ± 1019 a	11099 ± 359 a
Cu (μg/g DW)	18.6 ± 1.36 a	20.1 ± 1.81 a	18.7 ± 1.33 a
Mn (μg/g DW)	911.0 ± 72.8 a	934.9 ± 34.2 a	976.5 ± 78.3 a

Supplementary Table S2. *Translocation factor* (TF, ratio of metal content in young or mature leaf to rhizome) and *bioaccumulation factor* of the leaves (BAF, ratio of metal content in young or mature leaf to soil metal content) for the quantified elements in the present study. Data are means (n = 3) and for the lucidity of table, SD values are not shown. Values within rows (separately for young or mature leaves) followed by the same letter, are not significantly different according to Tukey's test (at P<0.05 level). Although Ni and Cd were applied as a foliar spray, TF and BAF values are comparable at least in control treatments with other species and other elements in all treatments. nd – not determined.

	young leaf			mature leaf		
translocation factor	control	Ni	Cd	control	Ni	Cd
Cd	0.424 b	nd	1.384 a	0.433 b	nd	2.091 a
Ni	0.049 b	0.094 a	nd	0.045 b	0.092 a	nd
K	3.535 a	4.162 a	3.814 a	2.349 a	2.807 a	2.258 a
Ca	0.264 a	0.265 a	0.253 a	1.472 a	1.601 a	1.525 a
Mg	0.291 a	0.317 a	0.369 a	0.974 b	0.961 b	1.345 a
Fe	0.022 a	0.021 a	0.016 a	0.031 a	0.029 a	0.030 a
Cu	1.059 a	1.024 a	0.973 a	0.485 a	0.341 b	0.261 b
Mn	0.083 a	0.077 a	0.081 a	0.119 a	0.084 a	0.092 a
bioaccumulation factor						
Cd	0.847 b	nd	9.302 a	0.873 b	nd	10.09 a
Ni	0.117 b	0.429 a	nd	0.108 b	0.427 a	nd
K	9.678 a	9.712 a	9.004 a	6.428 a	6.553 a	5.327 b
Ca	0.265 a	0.251 a	0.230 a	1.479 a	1.512 a	1.386 a
Mg	0.438 a	0.515 a	0.449 a	1.454 a	1.562 a	1.639 a
Fe	0.0038 a	0.0036 a	0.0025 b	0.0053 a	0.0051 a	0.0049 a
Cu	0.669 a	0.672 a	0.746 a	0.309 a	0.225 b	0.200 b
Mn	0.025 a	0.022 a	0.023 a	0.036 a	0.024 b	0.026 b

Supplementary Table S3. Accumulation of selected macronutrients and micronutrients in the fern *Adiantum raddianum* after 30 days of foliar application of exogenous Cd or Ni (1 µM solution). Data are means ± SDs shown as bars (n = 3). Values within column for each organ, followed by the same letter(s), are not significantly different according to Tukey's test (at P<0.05 level).

		K (mg/g DW)	Ca (mg/g DW)	Mg (mg/g DW)	Fe (µg/g DW)	Cu (µg/g DW)	Mn (µg/g DW)
young leaf	control	34.1 ± 3.27 a	0.68 ± 0.041 a	1.99 ± 0.41 a	40.4 ± 3.20 a	12.4 ± 1.76 a	22.9 ± 2.45 a
	Ni	35.0 ± 2.58 a	0.69 ± 0.026 a	2.23 ± 0.17 a	40.9 ± 4.08 a	13.8 ± 2.16 a	20.7 ± 3.02 a
	Cd	33.6 ± 2.44 a	0.66 ± 0.054 a	1.91 ± 0.23 a	28.7 ± 3.55 b	13.2 ± 1.51 a	22.3 ± 3.74 a
mature leaf	control	22.6 ± 3.35 a	3.84 ± 0.32 a	6.63 ± 0.50 a	56.3 ± 3.79 a	5.77 ± 0.20 a	32.8 ± 3.12 a
	Ni	23.7 ± 2.53 a	4.18 ± 0.17 a	6.76 ± 0.51 a	57.6 ± 3.27 a	4.53 ± 0.32 b	22.6 ± 2.86 b
	Cd	20.0 ± 1.48 a	3.96 ± 0.15 a	6.97 ± 0.34 a	54.9 ± 5.32 a	3.72 ± 0.39 b	25.0 ± 3.35 ab
rhizome	control	9.65 ± 0.81 a	2.63 ± 0.45 a	6.81 ± 0.50 a	1832.8 ± 277.7 a	12.0 ± 1.60 a	277.3 ± 34.2 a
	Ni	8.47 ± 1.13 a	2.67 ± 0.26 a	7.03 ± 0.26 a	1948.0 ± 257.5 a	13.4 ± 1.91 a	268.2 ± 22.0 a
	Cd	8.95 ± 0.97 a	2.61 ± 0.23 a	5.18 ± 0.22 b	1816.4 ± 273.9 a	14.5 ± 1.52 a	276.6 ± 30.3 a

Supplementary Table S4. Pearson's correlation analysis between parameters in the **young leaves** of the fern *Adiantum raddianum* after 30 days of foliar application of exogenous Cd or Ni (1 µM solution). * The corresponding correlation in R is considered significant as an off-diagonal element of P was smaller than the significance level of 0.05. Green and red numbers indicate significantly positive or negative values, respectively.

	AsA	GSH	PC2	TPC	flav.	FAA	prot.	SOD	CAT	K	Ca	Mg	Fe	Cu	Mn
AsA	1														
GSH	0.725*	1													
PC2	-0.925*	-0.903*	1												
TPC	0.112	-0.009	-0.109	1											
flav.	-0.056	-0.204	0.044	0.772*	1										
FAA	0.415	0.116	-0.299	0.524	0.445	1									
prot.	0.220	-0.190	-0.068	0.103	0.030	0.432	1								
SOD	0.220	-0.190	-0.068	0.103	0.030	0.432	1.000*	1							
CAT	-0.827*	-0.957*	0.949*	-0.091	0.063	-0.227	0.221	0.221	1						
K	0.340	0.020	-0.147	0.401	-0.012	0.016	0.294	0.294	-0.045	1					
Ca	0.247	0.548	-0.497	0.201	0.276	0.366	0.254	0.254	-0.446	-0.353	1				
Mg	0.306	0.322	-0.416	-0.007	0.286	0.578	0.327	0.327	-0.341	-0.594	0.790*	1			
Fe	0.914*	0.771*	-0.860*	0.066	-0.127	0.283	-0.052	-0.052	-0.840*	0.339	0.143	0.115	1		
Cu	-0.442	-0.411	0.533	0.390	0.260	0.381	-0.217	-0.217	0.370	0.016	-0.286	-0.214	-0.346	1	
Mn	-0.236	0.137	-0.022	0.336	0.072	-0.325	-0.268	-0.268	-0.076	-0.060	0.196	-0.188	-0.237	-0.108	1

Supplementary Table S5. Pearson's correlation analysis between parameters in the **mature leaves** of the fern *Adiantum raddianum* after 30 days of foliar application of exogenous Cd or Ni (1 μM solution). * The corresponding correlation in R is considered significant as an off-diagonal element of P was smaller than the significance level of 0.05. Green and red numbers indicate significantly positive or negative values, respectively.

	AsA	GSH	PC2	TPC	flav.	FAA	prot.	SOD	CAT	K	Ca	Mg	Fe	Cu	Mn
AsA	1														
GSH	0.957*	1													
PC2	-0.960*	-0.948*	1												
TPC	0.315	0.298	-0.300	1											
flav.	0.435	0.555	-0.386	0.310	1										
FAA	-0.875*	-0.879*	0.918*	-0.218	-0.257	1									
prot.	-0.924*	-0.939*	0.899*	-0.539	-0.529	0.778*	1								
SOD	-0.924*	-0.939*	0.899*	-0.539	-0.529	0.778*	1.000*	1							
CAT	0.661	0.622	-0.551	0.264	0.720*	-0.513	-0.555	-0.555	1						
K	0.654	0.645	-0.493	-0.123	0.461	-0.455	-0.504	-0.504	0.738*	1					
Ca	0.037	-0.001	-0.102	-0.285	-0.547	0.004	-0.065	-0.065	-0.639	-0.231	1				
Mg	-0.425	-0.370	0.249	-0.012	-0.401	0.300	0.224	0.224	-0.795*	-0.827*	0.636	1			
Fe	0.260	0.335	-0.142	0.000	-0.079	-0.235	-0.354	-0.354	-0.134	0.376	0.364	-0.073	1		
Cu	0.788*	0.769*	-0.721*	0.467	0.581	-0.664	-0.766*	-0.766*	0.568	0.394	-0.177	-0.370	0.174	1	
Mn	0.269	0.255	-0.307	0.537	0.384	-0.441	-0.267	-0.267	0.455	-0.146	-0.605	-0.183	-0.359	0.571	1

Supplementary Table S6. Pearson's correlation analysis between parameters in the **rhizome** of the fern *Adiantum raddianum* after 30 days of foliar application of exogenous Cd or Ni (1 µM solution). * The corresponding correlation in R is considered significant as an off-diagonal element of P was smaller than the significance level of 0.05. Green and red numbers indicate significantly positive or negative values, respectively.

	AsA	GSH	PC2	TPC	flav.	FAA	K	Ca	Mg	Fe	Cu	Mn
AsA	1											
GSH	0.586	1										
PC2	-0.256	-0.282	1									
TPC	0.086	0.067	-0.647	1								
flav.	-0.308	0.058	0.292	-0.082	1							
FAA	-0.031	0.263	0.763*	-0.718*	0.550	1						
K	-0.253	-0.480	-0.027	0.596	0.059	-0.455	1					
Ca	-0.038	-0.234	0.027	0.405	0.593	0.000	0.312	1				
Mg	0.286	0.432	-0.906*	0.689*	-0.291	-0.646	-0.122	0.047	1			
Fe	0.441	0.367	-0.204	-0.402	0.086	0.145	-0.435	-0.296	0.015	1		
Cu	-0.301	0.074	0.513	-0.025	0.801*	0.610	0.259	0.392	-0.438	-0.237	1	
Mn	-0.290	-0.300	-0.066	-0.083	0.474	0.127	-0.142	0.530	-0.028	-0.085	0.185	1

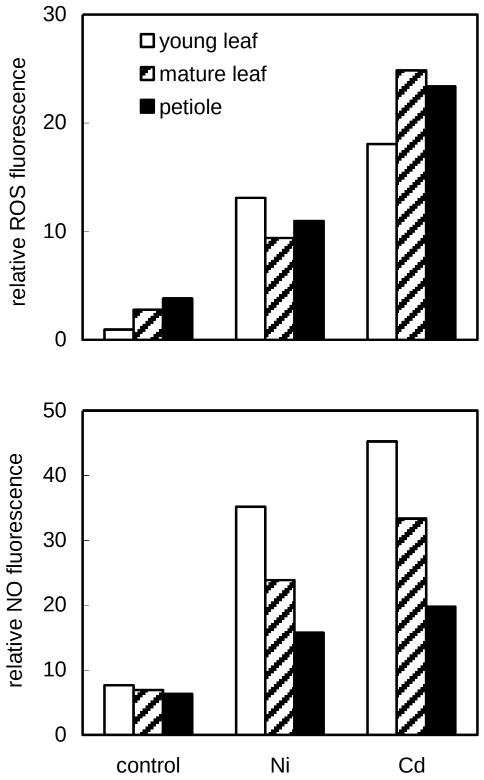

Supplementary Figure S1. Relative ROS and NO fluorescence from photos presented in the Fig. 2 in the main text. Note that the impact of Cd on the ROS formation was higher than the impact of Ni while the impact of Cd and Ni on the NO signal was similar in mature leaf and petiole.

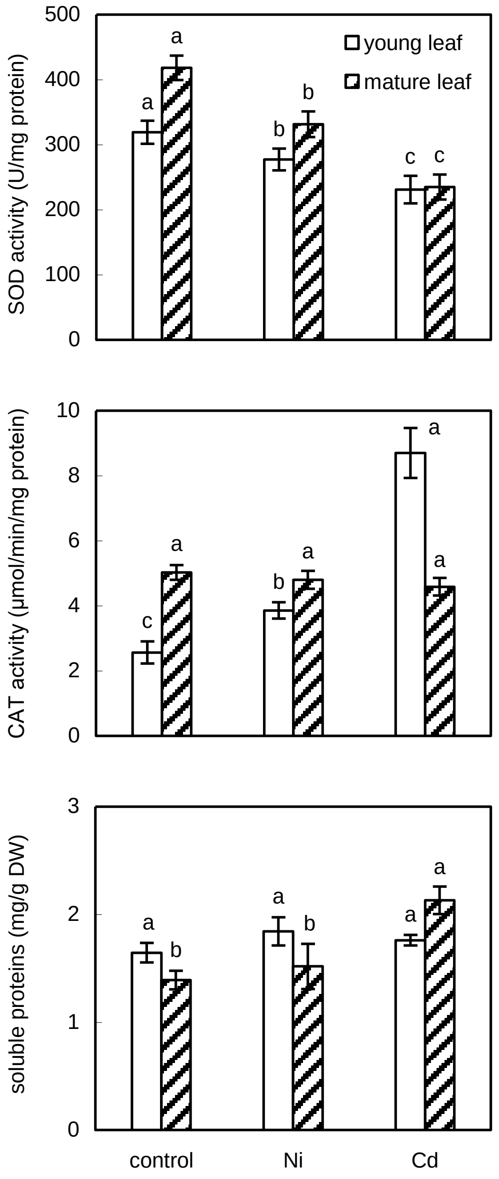

Supplementary Figure S2. Activities of superoxide dismutase (SOD) and catalase (CAT) and amount of soluble proteins in the fern *Adiantum raddianum* after 30 days of foliar application of exogenous Cd or Ni (1 μM solution). Control means plants without exogenous application of metals. Data are means ± SDs shown as bars (n = 3). Columns for young or mature leaves, followed by the same letter, are not significantly different according to Tukey's test (at $P<0.05$ level).

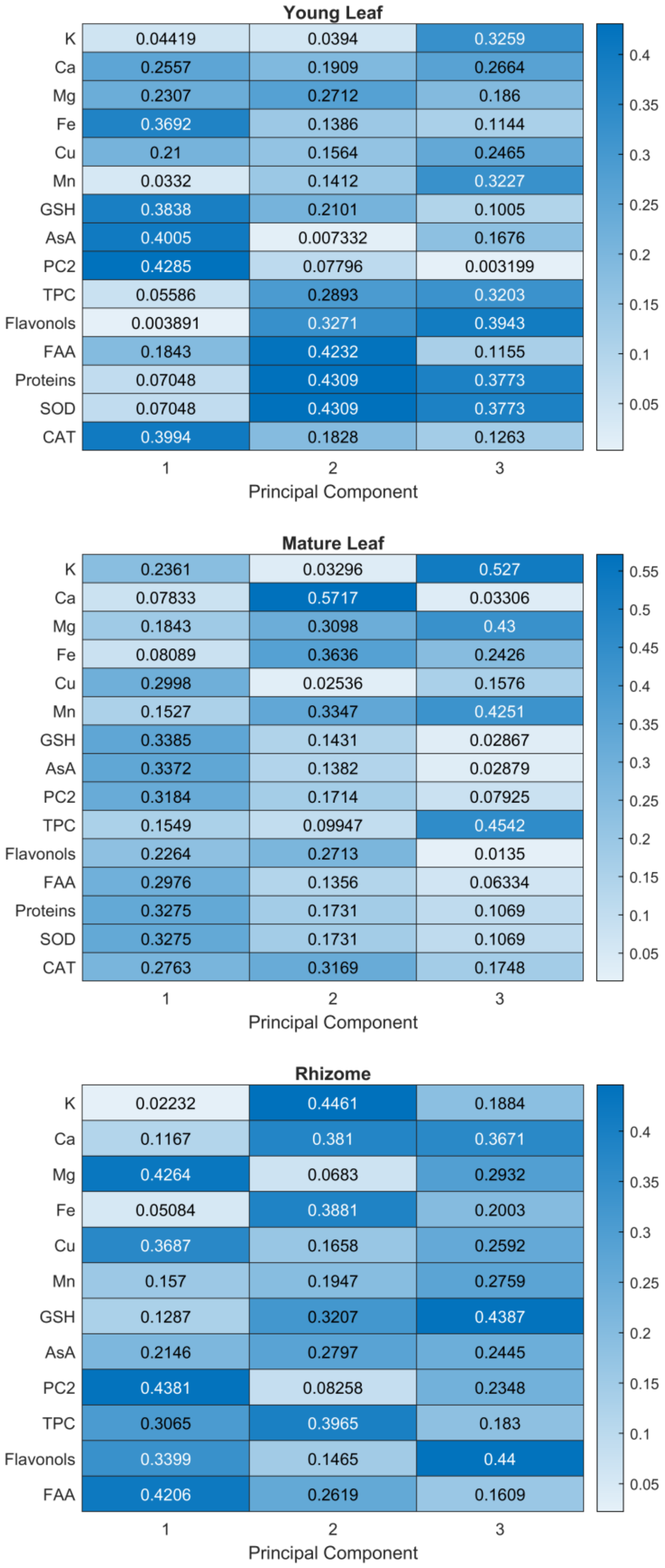

Supplementary Figure S3. Heat maps of the analyte loadings on the three components.

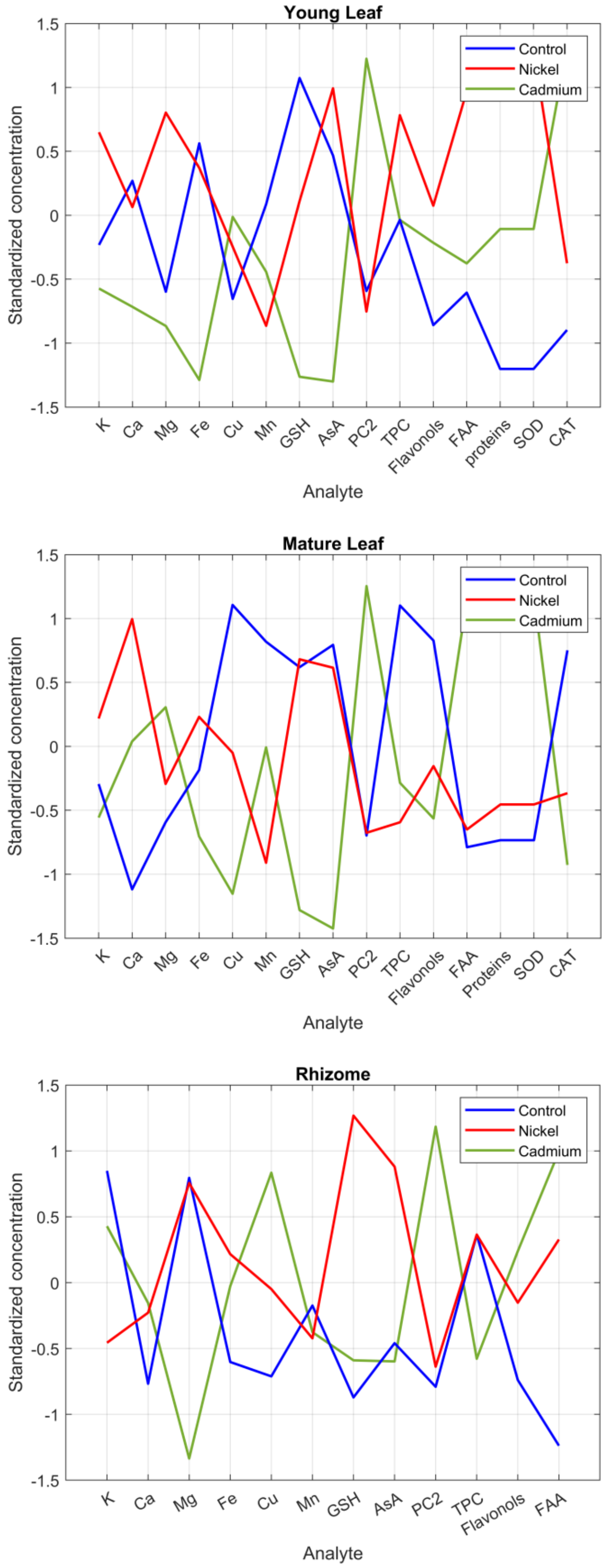

Supplementary Figure S4. Median values of standardized concentrations of individual analytes in different plant organs.
